# Microbial drivers of soil health: Integrating physical, chemical and biological properties for food security under climate change

**DOI:** 10.1016/j.crmicr.2026.100636

**Published:** 2026-06-23

**Authors:** Gloria Elva Dorado-González, Alberto Muro-Reyes, Sergio de los Santos-Villalobos, Daniel García-Cervantes, Jesús Gerardo García-Olivares, Héctor Manuel Robles-Berlanga, Héctor Gutiérrez-Bañuelos

**Affiliations:** a Unidad Académica de Medicina Veterinaria y Zootecnia, Universidad Autónoma de Zacatecas, Zacatecas, Mexico; b Departamento de Ciencias Agronómicas y Veterinarias, Instituto Tecnológico de Sonora, Ciudad Obregón, Sonora, Mexico; c Centro de Biotecnología Genómica, Instituto Politécnico Nacional, Reynosa, Tamaulipas, Mexico; d Independent Agricultural Consultant, Estado de Mexico, Mexico

**Keywords:** Soil health, Soil microbiome, Physical soil properties, Nutrient cycling, Climate stress, Soil resilience

## Abstract

Climate change is intensifying heat, drought/flooding extremes, salinity, and CO_2_-driven shifts that disrupt soil structure, chemistry, and biological activity, with cascading consequences for crop productivity and food security. This review synthesizes evidence that soil health results from the interconnected interactions among physical structure (aggregation, porosity, bulk density, and pore connectivity), chemical constraints (pH, salinity, nutrient availability, cation exchange capacity, and redox heterogeneity), and biological activity (microbial biomass, diversity, and functional pathways). A central conclusion is that climate impacts are frequently mediated through pore-scale microhabitats (oxygen and moisture gradients, redox microsites, and substrate accessibility), which reorganize microbial functional guilds and regulate C-N-P transformations, organic matter turnover, and aggregation dynamics. We highlight mechanistic pathways by which microbiomes actively shape soil resilience, including EPS/biofilm-mediated aggregate stabilization, extracellular enzyme systems that control depolymerization and nutrient acquisition, and metabolite-driven nutrient mobilization (e.g., organic acids and siderophores), alongside nitrogen and phosphorus cycling processes that are highly sensitive to aeration and moisture regimes. Evidence across agroecosystems indicates that effective climate-smart soil management is most robust when “habitat-first” practices (reduced disturbance, continuous plant inputs, organic amendments) are combined with context-dependent microbiome steering (diversified rotations/cover crops and targeted inoculants). Overall, integrating cross-domain indicators with mechanistic understanding offers actionable pathways to strengthen soil multifunctionality, stabilize yields under climate variability, and support sustainable food systems

## Highlights

• Climate stressors restructure soil microhabitats, reassembling microbial guilds that regulate C–N–P cycling and resilience.
• Cross-domain feedbacks link pore architecture and soil chemistry to microbiome functions and emergent soil health outcomes.
• Rhizosphere mechanisms (exudates, siderophores, ACC deaminase, ISR, AMF/N-fixing symbioses) provide actionable leverage points under stress.
• Integrative indicators connect physical, chemical, and biological metrics to soil multifunctionality for monitoring under climate extremes.
• Emerging tools (meta-omics, 3D imaging, spectroscopy, isotopic tracing, in situ sensors) enable mechanistic diagnosis and decision support.

## Graphical abstract

**Figure:**
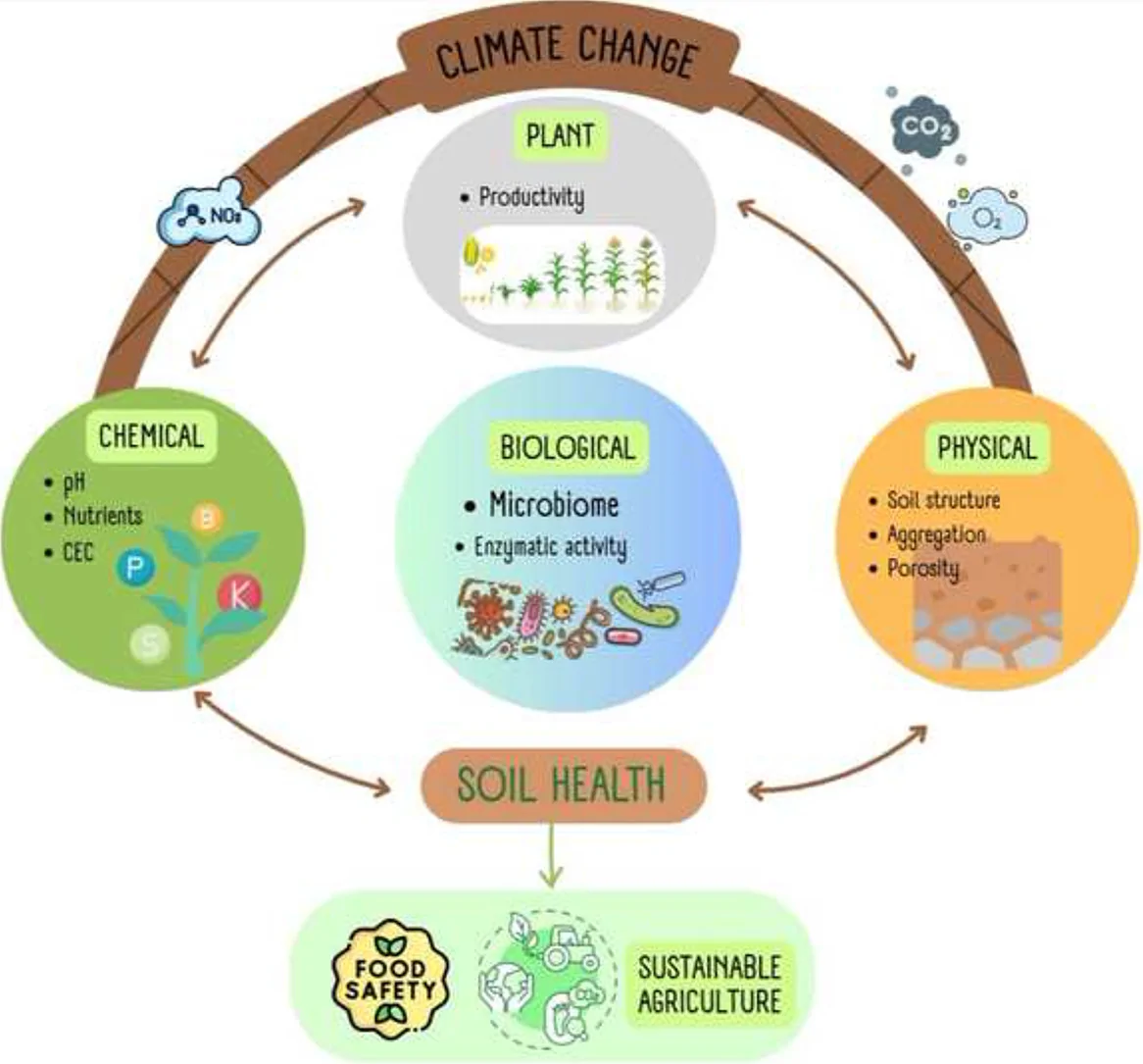

## 1 Introduction

Climate change is transforming agroecosystems worldwide through rising temperatures, altered precipitation patterns, and more frequent extremes, leading to cascading effects on soil water availability, nutrient cycling, crop productivity, and food security (IPCC, 2023; Laine and Leino, 2025). Since soil health underpins crop productivity and resilience, understanding how soils respond to climate variability has become a central component of climate-smart agriculture.

Soil health results from the combined interactions of physical, chemical, and biological domains rather than from any single property in isolation (Bünemann et al., 2018; Kibblewhite et al., 2008). Physical structure, including aggregation, porosity, bulk density, and pore connectivity, regulates water flow, gas exchange, and the spatial organization of microbial habitats. Chemical conditions, such as pH, salinity, nutrient availability, cation exchange capacity, and redox status, determine resource availability and physiological constraints for soil organisms. Biological activity, particularly microbial biomass, diversity, enzymatic activity, and functional pathways, mediates organic matter turnover, nutrient cycling, aggregation, and plant stress responses. These domains are linked by strong feedbacks: soil structure shapes oxygen and moisture availability, chemical gradients influence microbial community assembly and metabolism, and microbial activity in turn alters soil aggregation, nutrient mobilization, and organic matter dynamics (Young and Crawford, 2004; Totsche et al., 2018; Lehmann et al., 2020; Hartmann and Six, 2023).

Soil microorganisms are increasingly recognized not only as indicators of soil condition but also as active drivers of soil function. They decompose organic substrates, facilitate key steps of C-N-P cycling, and influence soil structure through extracellular polymeric substances (EPS), biofilms, enzymes, organic acids, siderophores, and other metabolites that affect particle binding, moisture retention, and nutrient mobilization (Costa et al., 2018; Zhang et al., 2024). Climate stressors such as heat, drought, salinity, and flooding can therefore reorganize microbial community composition and functional activity, with consequences for nutrient retention, greenhouse gas fluxes, soil resilience, and plant performance (Schimel et al., 2007; Evans and Wallenstein, 2014; Ramond et al., 2022; Bei et al., 2023; Ge and Wang, 2025).

Recent reviews have synthesized the transformative role of soil microbial communities in soil fertility, crop productivity, nutrient cycling, plant stress tolerance, and soil habitat modification (Iqbal et al., 2025; Philippot et al., 2024). However, these syntheses have not always integrated how climate stress acts through physical and chemical constraints at the microhabitat level, particularly when microbial taxa, functional traits, or general soil health indicators are considered separately (Bünemann et al., 2018; Lehmann et al., 2020; Hartmann and Six, 2023). Unlike traditional assessments that treat soil as a relatively homogeneous bulk matrix, this review emphasizes pore-scale microhabitats as a unifying framework for understanding how climate change reshapes soil health. In this view, climate stressors alter oxygen diffusion, moisture connectivity, redox microsites, and substrate accessibility, thereby reorganizing microbial guilds and regulating emergent soil functions (Young and Crawford, 2004; Totsche et al., 2018; Keiluweit et al., 2016; Rohe et al., 2021; Schlüter et al., 2025).

Here, we provide an integrative review that explicitly links climate stressors to: (i) physical and chemical shifts in soil, (ii) microbiome responses in community structure and functional pathways, and (iii) emergent soil functions relevant to food security. The novelty of this review lies in three aspects. First, it frames soil health responses to climate change through pore-scale microhabitats rather than through isolated physical, chemical, or biological indicators. Second, it connects microbial mechanisms such as EPS production, extracellular enzyme activity, nutrient mobilization, symbioses, and stress-response traits with measurable soil properties and management levers. Third, it discusses how this cross-domain understanding can be operationalized through habitat-first management and context-dependent microbiome steering to enhance soil resilience under climate stress.

**Fig. 1:**
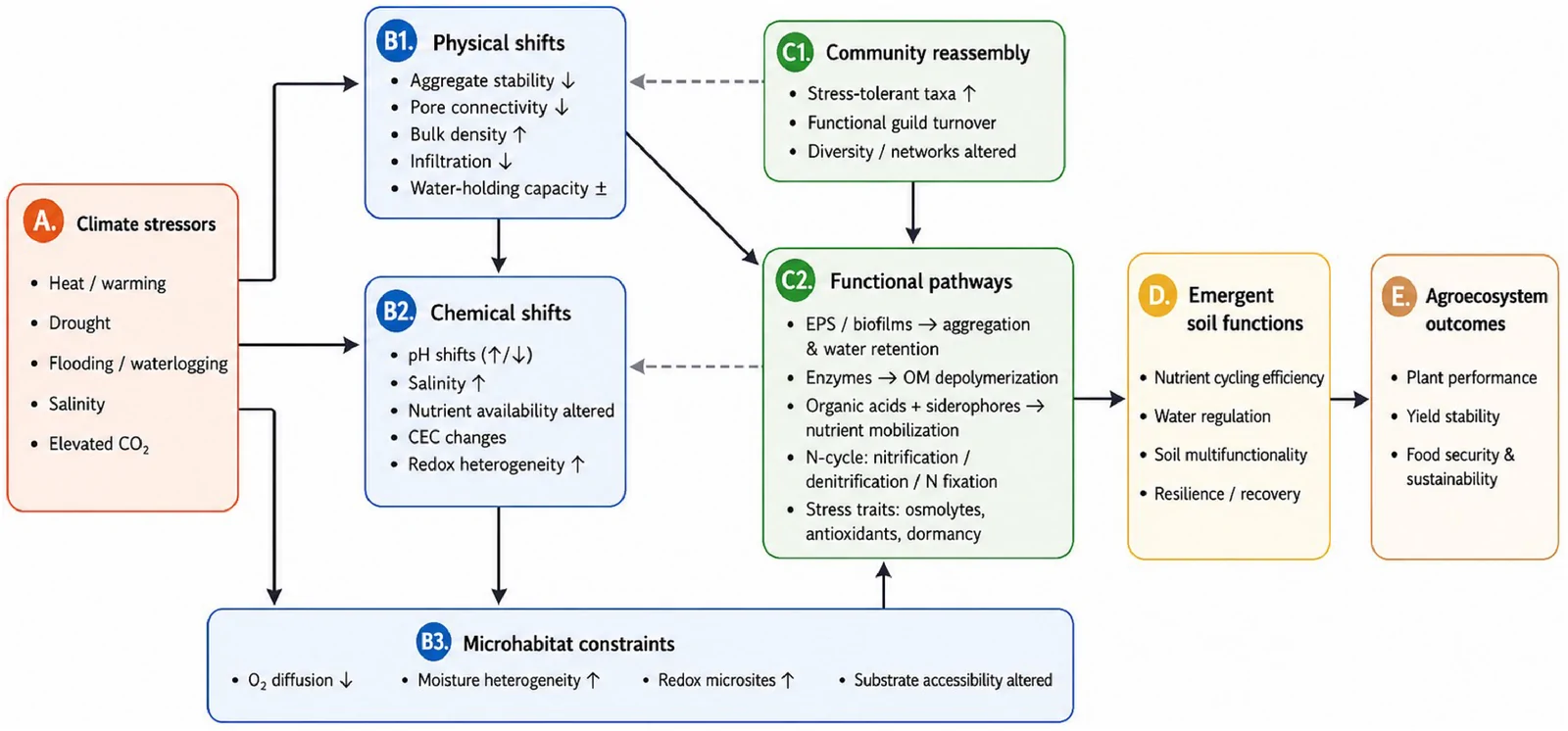
Mechanistic framework linking climate stressors, soil domain shifts, microbiome responses, and soil health outcomes. Solid arrows indicate dominant causal pathways; dashed arrows indicate feedback loops.

Fig. 1 summarizes the conceptual framework used throughout this review, linking climate stressors, soil domain shifts, microbiome responses, and soil health outcomes. This review does not aim to catalogue all existing soil health indicators or microbial taxa. Instead, it focuses on mechanistic linkages and integrative indicators that support monitoring and decision-making across agroecosystems, including systems affected by land degradation and climate variability. Although the review is globally oriented, semi-arid and degraded systems are highlighted when they provide useful examples for microbiome-informed soil restoration and climate adaptation. The remainder of the manuscript progresses from microbiome functions and plant–soil interactions to the physical, chemical, and management determinants of soil health, followed by management strategies, emerging tools, and future research needs.

## 2 The soil microbiome as a driver of ecosystem functions

Soil biological properties, especially microbiome-driven processes, are vital to agroecosystem resilience amid climate change because they control nutrient cycles, organic matter decomposition, soil aggregation, and plant stress responses. Instead of being passive indicators, microbial communities (including bacteria, archaea, fungi, protozoa, and viruses) actively influence soil functions through interacting functional groups and context-specific metabolic pathways (Delgado-Baquerizo et al., 2016; Banerjee et al., 2018; Fierer, 2017).

### 2.1 Functional guilds and mechanisms under climate stress

Climate stressors (heat, drought, salinity, and flooding) reshape oxygen and moisture regimes and reorganize microbial guilds controlling key biogeochemical processes. In nitrogen cycling, shifts in aeration and redox microsites can rebalance nitrification and denitrification potentials, with direct consequences for nitrogen retention and loss pathways (Ramond et al., 2022; Young and Crawford, 2004). At the physiological level, drought and heat can select for stress-tolerant strategies including antioxidant defense, heat shock responses, sporulation/dormancy, and cell-envelope remodeling, which in turn influence decomposition rates and nutrient release. For example, metatranscriptomic profiling of soils exposed to extreme summer drought showed increased expression of genes associated with oxidative stress protection (e.g., Ni-SOD), chaperones (GroES/GroEL), cell-wall biosynthesis, and sporulation, consistent with community-level functional reprogramming under abiotic stress (Bei et al., 2023; Ramond et al., 2022). These adaptive traits underpin plant–microbiome partnerships that enhance crop tolerance to drought and salinity, offering opportunities for microbiome engineering in climate-smart agriculture (Ge and Wang, 2025).

Beyond bacteria, fungi, archaea, and protists, soil viruses are increasingly recognized as active but still underexplored regulators of microbial community dynamics and biogeochemical cycling (Emerson, 2019; Graham et al., 2024). Viruses can influence soil functions through several mechanisms, including host cell lysis, changes in microbial mortality and turnover, horizontal gene transfer, and the carriage of auxiliary metabolic genes that may alter host metabolism during infection (Emerson, 2019; Sun et al., 2023). These processes can redirect carbon and nutrient flows through the viral shunt and may affect microbial pathways involved in C, N, P, and S cycling (Emerson, 2019; Sun et al., 2023; Graham et al., 2024). Recent stable-isotope probing combined with metagenomics provided evidence that soil viruses can participate in nitrogen cycling by infecting active diazotrophs and carrying auxiliary nitrogen-cycling genes, suggesting that viral pathways may contribute to nitrogen turnover under changing soil conditions (Liu et al., 2024). Therefore, climate-driven shifts in moisture, oxygen availability, and redox heterogeneity may not only reorganize bacterial and fungal guilds but also alter virus–host interactions that regulate nutrient cycling, microbial turnover, and soil resilience (Liu et al., 2024; Graham et al., 2024).

### 2.2 From diversity to function: indicators and multi-omics

Because microbial communities respond quickly to changes in temperature, moisture, salinity, and management, microbiome-sensitive indicators can provide early warnings of soil degradation or recovery. In practice, diversity is commonly quantified using alpha diversity (richness, Shannon), beta diversity, and network metrics (connectivity/modularity), while functional status is tracked using microbial biomass C/N, respiration (including qCO_2_), and enzyme activities such as β-glucosidase, phosphatase, and dehydrogenase, complemented by functional genes (e.g., amoA, nirK/nirS, nosZ, nifH, phoD) when pathway-level resolution is needed (Schloter et al., 2018; Nikitin et al., 2022). Additional community diagnostics include phospholipid fatty acid (PLFA) profiles and DNA-based methods that capture shifts in community structure relevant to soil functioning (Bünemann et al., 2018; Schloter et al., 2018). Interpretation is context-dependent and is strengthened by standardized frameworks that link indicators to constraints and functions across soil types and climates (Nikitin et al., 2022). These indicators are particularly informative under drought–rewetting cycles, salinity stress, and compaction/disturbance gradients (e.g., no-till vs conventional tillage; amended vs non-amended systems), where shifts in oxygen/moisture microhabitats translate into measurable changes in nutrient cycling efficiency, aggregation/water regulation, and resilience.

Recent advances in metagenomics and metatranscriptomics enable functional inference and pathway-level assessment, helping shift from correlative diversity metrics toward a mechanistic understanding of how microbial processes mediate soil functions under climate stress (Fierer, 2017; Bei et al., 2023). Integrating biological indicators with physical and chemical measurements supports cross-domain soil health assessments and informs the indicator-based synthesis summarized in Table 1.

**Table 1:** Core soil health domains, representative indicators, and microbiome-mediated links across physical, chemical, and biological properties.

Soil health domain	Representative indicators (examples)	Microbiome-mediated links (mechanisms/examples)	Emergent soil functions supported	Climate-stress sensitivity (typical direction)	References
Physical: Structure & water regulation	Aggregate stability; bulk density; macro/microporosity; infiltration; plant-available water	EPS/biofilms and fungal hyphae promote aggregation/pore continuity; microbial residues contribute to stable aggregates; pore-scale constraints shape microbial function	Water buffering; aeration; root penetration; yield stability	**Drought/heat:** connectivity often ↓; heterogeneity ↑; **flooding:** structure vulnerability + redox heterogeneity ↑	Six et al., 2004; Totsche et al., 2018; Costa et al., 2018; Hartmann and Six, 2023
Physical: Soil water dynamics	Soil moisture variability; hydraulic conductivity; field capacity; plant-available water; evaporation losses	EPS and biofilms increase water retention near cells/roots; rhizosphere activity modulates wetting–drying behavior and local hydrophobicity; mycorrhizal networks improve plant water uptake	Water buffering; drought resilience; yield stability	Drought: moisture heterogeneity ↑; plant-available water ↓; activity shifts toward stress tolerance	Franzluebbers, 2002; Betz et al., 1998; Costa et al., 2018; Smith and Smith, 2011; Hartmann and Six, 2023
Chemical: pH & buffering capacity	pH; buffering capacity; carbonate content; Al³⁺ toxicity risk	pH structures bacterial communities and functional expression; shifts in community composition/PLFA; enzyme systems respond to pH constraints	Nutrient availability; decomposition efficiency; biological functioning	**Heat/drought:** pH effects amplified via diffusion limits; **salinity:** pH–ionic interactions alter assembly	Rousk et al., 2010; Lauber et al., 2009; Bartram et al., 2014
Chemical: Salinity & ionic strength	EC; Na⁺; SAR; Cl⁻; osmotic potential	Salinity/sodicity reduces microbial activity; selects tolerant communities; disrupts rhizosphere signaling and plant–microbe interactions	Establishment and nutrient cycling under osmotic stress	**Salinity:** diversity/activity often ↓; stress traits ↑; nutrient-use efficiency ↓ unless managed	Rietz and Haynes, 2003; Rodríguez-Eugenio et al., 2018; Lugtenberg and Kamilova, 2009
Chemical: Nutrient pools & stoichiometry	NH₄⁺/NO₃⁻; available P; C:N:P ratios; micronutrients	Microbial nutrient cycling regulates pools/losses; organic acids and microbial solubilization support P availability; rhizosphere processes improve fertilizer-use efficiency	Fertility; nutrient-use efficiency; productivity	**Drought:** diffusion-limited supply; **flooding:** N losses ↑; **heat:** turnover may ↑ then decline	Burgin and Hamilton, 2007; Cleveland and Liptzin, 2007; Jones, 1998; Adesemoye and Kloepper, 2009; García-Berumen et al., 2024; Ramond et al., 2022
Chemical: Redox conditions	Eh; dissolved O₂; Fe/Mn speciation; GHG flux proxies	Aerobic/anaerobic microsites regulate N transformations; self-organization at pore scale creates biogeochemical hotspots; extremes shift pathways	Regulation of N losses; C stabilization vs emissions; resilience	**Flooding:** O₂ ↓, Eh ↓; **rewetting:** pulse emissions possible	Keiluweit et al., 2016; Rohe et al., 2021; Young and Crawford, 2004
Biological: Biomass & activity	Microbial biomass C/N; respiration; qCO₂; substrate-induced respiration	Stress-response physiology shifts efficiency and activity; extremes alter strategies; activity integrates resource supply + stress	Decomposition; fertility maintenance; multifunctionality	**Drought/heat:** activity often ↓ (rewetting pulses possible); **extremes:** efficiency ↓	Schimel et al., 2007; Evans and Wallenstein, 2014; Bei et al., 2023; Nikitin et al., 2022
Biological: Diversity & community structure	Alpha/beta diversity; network connectivity; fungal:bacterial ratio	Diversity supports multifunctionality; keystone taxa structure communities and function; shifts affect stability and disease suppression	Resilience; stability; disease suppression potential	**Chronic stress:** diversity ↓; recovery depends on habitat quality/management	Delgado-Baquerizo et al., 2016; Banerjee et al., 2018; Wagg et al., 2014; Bastida et al., 2021
Biological: Functional potential & enzymes / meta-omics	Enzymatic activity; functional potential (meta-omics); indicators of soil ecological functions	Microbial functions are better predictors of soil ecological functions than taxonomy alone; metagenomics, metatranscriptomics, and viral metagenomics reveal functional potential, active pathways, and virus-host interactions; soil viruses may influence microbial turnover and C—N-P cycling through lysis and auxiliary metabolic genes.	Adaptive capacity; nutrient cycling efficiency	**Drought/heat:** functional expression shifts; **extremes:** pathway turnover	Burns et al., 2013; Fierer, 2017; Nikitin et al., 2022; Sun et al., 2023; Graham et al., 2024; Liu et al., 2024; Bei et al., 2023
Management leverage (microbiome steering)	SOC/OM inputs; tillage intensity; cover crops/rotations; inoculants	Management reshapes habitat/resources → microbiome structure/function; harnessing plant microbiomes and PGPR improves resilience and nutrient efficiency	Long-term soil health; productivity; sustainability	**Degraded systems:** improvements show time-lags; context-dependence high	Bünemann et al., 2018; Lehmann et al., 2020; Busby et al., 2017; Compant et al., 2019; Ge and Wang, 2025

These microbiome mechanisms are strongly conditioned by plant-soil feedbacks and management practices, which together determine whether soils sustain function or shift toward degradation under climate extremes.

## 3 Microbiome interactions with plants and soils under climate stress

Plant-microbiome interactions in the rhizosphere are key determinants of how agroecosystems respond to climate stress because they regulate resource acquisition, root development, and stress signaling at the soil-plant interface. Climate extremes such as drought, heat, salinity, and flooding reshape rhizosphere microhabitats (moisture and oxygen gradients, nutrient diffusion, and redox conditions), thereby shifting microbial community structure and function (Schimel et al., 2007; Evans and Wallenstein, 2014; Bei et al., 2023). From a microbial-sciences perspective, it is useful to frame these interactions in terms of functional guilds and representative taxa: for example, rhizosphere-enriched bacteria such as *Pseudomonas, Bacillus*, and *Streptomyces*; associative nitrogen fixers such as *Azospirillum*; symbiotic diazotrophs such as *Rhizobium*/*Bradyrhizobium*; and arbuscular mycorrhizal fungi (AMF; e.g., *Rhizophagus*/*Glomus*) frequently contribute traits linked to nutrient mobilization, hormone modulation, and disease suppression under stress (Lugtenberg and Kamilova, 2009; Mendes et al., 2011; Compant et al., 2019; Smith and Smith, 2011). This mechanistic focus helps identify actionable leverage points for climate-smart agriculture (Busby et al., 2017; Compant et al., 2019).

### 3.1 Root exudates as drivers of rhizosphere assembly and nutrient mobilization

Root exudates supply the main carbon and energy inputs that shape rhizosphere microbiomes, and their composition can change under stress, affecting microbial recruitment and function. Low-molecular-weight organic acids (e.g., citrate, malate, oxalate) help mobilize nutrients that are otherwise sparingly available through chelation and pH micro-shifts, impacting both microbial activity and plant nutrient uptake (Jones, 1998). During drought or nutrient shortages, plants often adjust exudation patterns and rhizodeposition, favoring microbial guilds capable of utilizing diverse substrates or producing metabolites that improve nutrient availability and stress tolerance (Compant et al., 2019; Busby et al., 2017). In semi-arid environments, microbiome-driven phosphorus solubilization has been recognized as an effective means of supporting nutrient acquisition under water stress, linking rhizosphere processes to sustainable nutrient management (García-Berumen et al., 2024).

### 3.2 Siderophores and nutrient acquisition traits under stress

Iron and trace elements often constrain plant and microbial metabolism in aerobic soils, and these constraints can be exacerbated when drought reduces diffusion and rhizosphere hydration. Many plant-associated bacteria (often including *Pseudomonas* and *Bacillus*) produce siderophores—high-affinity Fe-chelating compounds—that increase Fe bioavailability and indirectly suppress pathogens through competitive exclusion (Lugtenberg and Kamilova, 2009; Compant et al., 2019). Beyond iron acquisition, siderophore-producing rhizobacteria may contribute to competitive dynamics that help stabilize beneficial community functions under stress, especially when combined with other plant-growth-promoting traits in rhizosphere consortia (Adesemoye and Kloepper, 2009).

### 3.3 ACC deaminase and phytohormone modulation under abiotic stress

Abiotic stress often raises plant ethylene levels, which can hinder root growth and decrease nutrient and water uptake. A well-known microbial mechanism that counters this effect is the production of 1-aminocyclopropane-1-carboxylate (ACC) deaminase, which reduces ethylene production by breaking down the ethylene precursor ACC. Along with microbial auxin production and other hormonal influences, this process can encourage root development, improve root structure, and help plants better acquire resources during drought and salinity stress (Lugtenberg and Kamilova, 2009; Adesemoye and Kloepper, 2009; Compant et al., 2019). Associative rhizobacteria and endophytic communities enriched in stressed plants (often including genera reported in plant microbiome reviews such as *Azospirillum, Pseudomonas*, and *Bacillus*) provide representative examples of microbial traits that connect rhizosphere signaling to plant-scale outcomes (Compant et al., 2019; Busby et al., 2017).

### 3.4 Disease suppression and induced systemic resistance (ISR)

Climate stress can raise plant vulnerability to disease by weakening defenses and shifting the balance between beneficial and harmful microbes. Disease-suppressive soils serve as clear examples of how microbiomes support plant health: specific rhizosphere communities can fight pathogens through antibiosis and competition and by boosting plant defenses, leading to induced systemic resistance (ISR) (Mendes et al., 2011; Lugtenberg and Kamilova, 2009). Reported suppressive microbiomes often combine bacterial antagonists (frequently including *Pseudomonas* spp. and actinobacterial groups such as *Streptomyces*) with fungal contributors (e.g., beneficial saprotrophs and mycoparasites highlighted in plant microbiome syntheses), supporting the concept that community composition and functional redundancy are critical for stability under heat and moisture extremes (Busby et al., 2017; Compant et al., 2019).

### 3.5 Symbioses supporting stress tolerance and nutrient acquisition

Arbuscular mycorrhizal fungi (AMF) enhance nutrient acquisition and can improve plant water relations through both plant-mediated and soil-mediated mechanisms (Smith and Smith, 2011; Augé et al., 2015). At the plant level, AMF may alter root hydraulic properties by improving root architecture, aquaporin regulation, and the effective absorptive interface for water and nutrient uptake (Augé et al., 2015). At the soil and rhizosphere levels, extraradical hyphae extend beyond the root depletion zone, increase the volume of soil explored, and improve access to water and phosphorus under diffusion-limited conditions (Smith and Smith, 2011; Hartmann and Six, 2023). Hyphal networks also contribute to soil aggregation by physically enmeshing particles and promoting microhabitats where microbial residues and extracellular polymeric substances stabilize pores and water films (Rillig et al., 2015; Hartmann and Six, 2023). Thus, AMF effects on drought tolerance should not be interpreted only as changes in bulk soil hydraulic conductivity, but as combined effects on root hydraulic conductance, rhizosphere water retention, pore connectivity, nutrient diffusion, and plant water-use performance (Smith and Smith, 2011; Augé et al., 2015; Rillig et al., 2015; Hartmann and Six, 2023).

These mechanisms are particularly relevant in low-input and dryland systems, where water and phosphorus are often simultaneously limiting. Representative AMF groups commonly discussed in the literature, such as Rhizophagus/Glomus, illustrate how hyphal foraging can increase the effective soil volume explored and improve phosphorus acquisition under drought-induced diffusion constraints (Smith and Smith, 2011; Hartmann and Six, 2023). Under climate stress, AMF-associated benefits often manifest as improved plant nutrient status, water-use efficiency, and growth stability rather than as a single hydraulic response (Augé et al., 2015). Importantly, these symbioses scale from the root interface to soil functioning: improved nutrient capture and rhizosphere carbon inputs can support microbial activity and enzyme-mediated nutrient cycling, while hyphal networks and rhizosphere structuring contribute to aggregation and water regulation—two functions repeatedly linked to resilience under drought and heat (Rillig et al., 2015; Hartmann and Six, 2023; Lehmann et al., 2020).

Nitrogen-fixing partnerships provide another key symbiotic pathway under stress. Symbiotic diazotrophs associated with legumes (e.g., *Rhizobium*/*Bradyrhizobium*) and actinorhizal associations (e.g., *Frankia* in relevant hosts) are well-established functional groups that can sustain N supply when mineral N availability is constrained or nutrient diffusion is limited during drought (Adesemoye and Kloepper, 2009; Compant et al., 2019). In addition, free-living and associative diazotroph guilds (reported broadly across plant microbiome syntheses) can contribute to N inputs and interact with plant signaling and root architecture responses under stress (Compant et al., 2019). Together, AMF and diazotrophic partnerships illustrate how symbioses buffer plants against climate-driven resource stress by coupling root-scale processes (water and nutrient acquisition) with microbiome functions central to soil fertility and resilience.

Interactions between AMF and nitrogen-fixing guilds under drought may be synergistic or competitive, depending on resource availability, host plant demand, and microhabitat conditions (Hodge and Storer, 2015; Compant et al., 2019). Synergy can occur when AMF improve phosphorus and water acquisition, thereby supporting the high energy and phosphorus requirements of biological nitrogen fixation in symbiotic or associative diazotrophs (Smith and Smith, 2011; Hodge and Storer, 2015). However, competition may arise because AMF, free-living diazotrophs, and other rhizosphere microorganisms depend on plant-derived carbon and occupy overlapping rhizosphere microsites. Under drought, reduced carbon allocation, limited nutrient diffusion, and heterogeneous water films may intensify competition for root exudates, phosphorus, and microsite access, potentially shifting the balance among AMF, associative diazotrophs, and free-living N-fixers (Compant et al., 2019; Ramond et al., 2022). Therefore, the net contribution of mycorrhizal and diazotrophic partnerships to drought resilience should be interpreted as context-dependent rather than universally additive, and future studies should evaluate these guilds together using functional genes, isotopic N tracing, root colonization, and rhizosphere microhabitat measurements (Hodge and Storer, 2015; Ramond et al., 2022).

### 3.6 Implications for microbiome-informed interventions under climate stress

Mechanistic pathways, including exudate-mediated recruitment, siderophore production, ACC deaminase activity, ISR priming, and mycorrhizal/diazotrophic symbioses, offer actionable targets for microbiome-informed management. However, effectiveness is context-dependent because soil structure, chemistry, and microhabitats constrain microbial establishment and activity. Therefore, interventions such as tailored bioinoculants, organic amendments, and management practices that stabilize rhizosphere habitats should be viewed as strategies to enhance the functional expression and persistence of beneficial traits, rather than as universal solutions (Busby et al., 2017; Compant et al., 2019; García-Berumen et al., 2024).

## 4 Determinants of soil health under climate change

Climate change alters soil health through interacting physical, chemical, and biological factors that operate across scales, from pore-level microhabitats to ecosystem-level processes. Warming, changing precipitation patterns, and extreme events affect soil structure and water movement, shift chemical gradients (such as pH, salinity, nutrient availability, and redox conditions), and reorganize microbiome composition and functional pathways that control C-N-P cycling and aggregation. Because these domains are connected by feedback loops, understanding their mechanistic links is crucial for identifying indicators and management strategies that improve soil resilience and sustain agroecosystem productivity under climate stress (Kibblewhite et al., 2008; Bünemann et al., 2018; Lehmann et al., 2020; Totsche et al., 2018; Young and Crawford, 2004).

### 4.1 Physical determinants: structure, porosity, and water dynamics

In agroecosystems affected by climate change, soil physical structure greatly limits microbial habitats and their ability to support plant growth and food security. Texture, aggregation, pore connectivity, and bulk density control air and water movement, as well as the spatial distribution and accessibility of substrates for microbial communities. These physical constraints create pore-scale microhabitats, influence oxygen diffusion, water-film thickness, redox microsites, and substrate accessibility, which in turn select for specific functional guilds and regulate rates of nitrification, denitrification, decomposition, and aggregate formation (Young and Crawford, 2004; Totsche et al., 2018). Conversely, microorganisms influence soil physical properties by producing extracellular polymeric substances (EPS), forming biofilms, and generating microbial residues that help stabilize aggregates and enhance water retention and structural integrity under climate stress (Costa et al., 2018; Lehmann et al., 2020).

Texture and aggregation act as habitat filters. Soil texture (sand, silt, and clay proportions) influences water movement and sets the physical environment for microbial activity. Fine-textured soils tend to hold more water because of smaller pores, but limited aeration can create anaerobic microsites that support redox-sensitive processes and taxa. Coarse-textured soils drain quickly and usually have lower water and nutrient retention, which can decrease microbial biomass and activity during drought (Six et al., 2004). In addition to texture, soil structure describes how particles clump into aggregates, forming macro- and micropore networks. Well-aggregated soils enhance aeration, root growth, and water storage, while also offering microhabitats that shield microorganisms from temperature and moisture changes (Bronick and Lal, 2005; Chenu and Cosentino, 2011). Aggregates also serve as sites for organic matter buildup and enzyme activity, boosting microbial growth and function and helping maintain stable soil structure (Chenu and Cosentino, 2011; Totsche et al., 2018). Microbial extracellular polymeric substances (EPS) and biofilms can further stabilize aggregates, increasing soil resilience against structural damage during extreme climatic conditions (Costa et al., 2018; Lehmann et al., 2020).

Porosity, bulk density, and oxygen diffusion. Porosity and pore-size distribution regulate oxygen availability, water retention, and microbial mobility. Differences between macropores and micropores are especially relevant under climate variability because they determine gas exchange, hydration continuity, and refuge habitats during drying (Golparvar et al., 2021). Bulk density reflects many structural constraints: when it increases due to machinery traffic, intensive tillage, or livestock trampling, porosity and pore connectivity decrease, limiting oxygen diffusion and root penetration while suppressing microbial activity (Hamza and Anderson, 2005; Beylich et al., 2010). Because nitrification, denitrification, and decomposition depend on oxygen diffusion and water-film thickness, compaction-driven changes in the pore network can alter biogeochemical pathways and reduce soil functional resilience (Beylich et al., 2010; Young and Crawford, 2004). Over time, microbial biofilms and EPS can help stabilize aggregates and partially restore pore continuity, but the extent of this feedback depends on organic inputs and the disturbance regime (Costa et al., 2018; Lehmann et al., 2020).

Water dynamics, drought stress, and functional pulses. Water availability is a key regulator of microbial activity, nutrient diffusion, and enzymatic processes. However, microbial responses to moisture are nonlinear and depend on functional groups, with extreme events like drought, flooding, and rewetting capable of reorganizing communities and shifting activity toward stress-tolerant or anaerobic taxa (Schimel et al., 2007; Evans and Wallenstein, 2014). Soils with higher organic matter content and stable aggregates tend to retain more plant-accessible water and create buffered microsites that support microbial survival and functional persistence during dry periods (Franzluebbers, 2002; Lehmann et al., 2020). Beyond total water content, the range of soil moisture that best supports plant growth depends on the balance among aeration, compaction, and matric potential. The Least Limiting Water Range (LLWR) combines these factors by identifying the moisture window where limitations are minimized. Long-term tillage and machinery traffic can narrow this window by increasing bulk density and reducing pore connectivity, thereby restricting microbial activity, root growth, and productivity (Betz et al., 1998; Hamza and Anderson, 2005).

Management effects on physical habitat quality. Agricultural practices deeply influence soil physical quality and microbial habitat structure. Conventional tillage breaks up aggregates and reduces pore continuity, raising erosion risk and limiting the stability of microbial habitats. Conversely, conservation practices such as no-till, cover cropping, and organic amendments tend to improve aggregation, porosity, and water retention (Blanco-Canqui and Lal, 2007; Franzluebbers, 2002). Restoring compaction and structural deterioration may require physical interventions—such as alleviating compacted layers and rebuilding organic matter—alongside strategies that promote soil-building microbial processes (Lehmann et al., 2020). For example, long-term conventional tillage has been linked to increased bulk density and decreased aggregate stability in semi-arid cropping systems, impairing infiltration and root penetration and thereby degrading microbial habitats and soil resilience (Rivera et al., 2000). Overall, enhancing soil physical habitat quality is essential for maintaining microbiome functions and ensuring productivity amid climate variability (Lehmann et al., 2020; Ge and Wang, 2025; García-Berumen et al., 2024).

### 4.2 Chemical determinants: pH, salinity, nutrient availability, and cation exchange capacity

Chemical conditions influence soil microbiome assembly and activity by shaping resource availability, toxicity limits, and reaction rates at the soil-water interface. Under climate change, shifts in rainfall patterns, warming, and extreme weather events alter solute concentrations, redox conditions, and nutrient transport, creating chemical gradients that interact with the pore network to determine microhabitats and regulate microbial metabolism (Young and Crawford, 2004; Totsche et al., 2018; Lehmann et al., 2020). Among chemical factors, soil pH, salinity/ionic strength, nutrient pools and ratios, and cation exchange capacity (CEC) strongly control microbial community structure, enzyme function, and plant nutrient uptake, thereby affecting soil multifunctionality and resilience in agroecosystems (Kibblewhite et al., 2008; Bünemann et al., 2018). pH is a key factor in microbial assembly and function. Soil pH is one of the strongest predictors of bacterial community composition and diversity across large spatial scales, reflecting its effects on nutrient solubility, metal toxicity, and enzyme activity (Lauber et al., 2009; Rousk et al., 2010; Bartram et al., 2014). Mechanistically, pH influences microbial physiology and controls the availability of substrates and electron acceptors, which affects decomposition and nutrient cycling. For example, pH-driven changes in community structure are often linked to shifts in functional expression, including enzymes that break down organic matter and pathways related to nitrogen transformations (Rousk et al., 2010). Since climate extremes can increase chemical heterogeneity (e.g., through drying-driven concentrations of solutes or rewetting-induced mobilization pulses), pH effects often interact with diffusion limitations and pore-scale microhabitats, influencing where and how microbes function (Young and Crawford, 2004; Totsche et al., 2018).

Salinity and ionic strength: osmotic stress and microbial efficiency. Salinity and sodicity impose osmotic and anionic constraints that can reduce microbial activity, alter carbon-use efficiency, and restructure rhizosphere interactions critical to plant performance. Irrigation-induced salinity and sodicity have been linked to declines in microbial activity and shifts in decomposition and nutrient cycling patterns, consistent with microbial reallocation toward stress tolerance rather than growth (Rietz and Haynes, 2003). Under drought, solute concentrations often rise as soils dry, intensifying osmotic stress; under erratic rainfall, rapid wetting can redistribute salts and create short-term chemical shocks. These dynamics can weaken beneficial plant-microbe interactions and reduce nutrient-use efficiency under climate stress, especially in salt-prone agroecosystems (Rietz and Haynes, 2003; Lugtenberg and Kamilova, 2009).

Nutrient availability and stoichiometry: linking C-N-P cycling to climate variability. Nutrient pools and stoichiometric constraints shape microbial growth strategies, enzyme allocation, and competition with plants for limiting resources. Microbial biomass often reflects characteristic C:N:P ratios, and deviations in substrate stoichiometry can shift microbial immobilization versus mineralization, thereby regulating nutrient supply to crops (Cleveland and Liptzin, 2007). Climate stressors modulate these processes by altering diffusion, moisture connectivity, and temperature-dependent reaction rates, which collectively control substrate access and microbial turnover (Schimel et al., 2007; Young and Crawford, 2004). In phosphorus-limited systems, microbial processes that mobilize P-such as phosphatase activity and the release of organic acids—can become especially important under drought because reduced moisture restricts diffusion and plant access to sparingly available P pools (Jones, 1998; García-Berumen et al., 2024). These mechanisms connect nutrient cycling directly to resilience: when microbial functional pathways maintain N and P availability during stress, agroecosystems are more likely to sustain productivity and stabilize yields (Adesemoye and Kloepper, 2009; García-Berumen et al., 2024).

Organic matter chemistry, CEC, and nutrient retention. Soil organic matter contributes to nutrient supply and chemical buffering by providing exchange sites that retain cations, supporting aggregation, and regulating microbially accessible carbon pools. CEC measures the capacity of soil colloids (clays and organic matter) to hold exchangeable cations, thereby influencing nutrient storage, leaching losses, and fertility management outcomes (Sparks, 2003). Climate-driven changes in organic matter inputs and decomposition can shift the balance between particulate and mineral-associated organic matter, affecting nutrient retention and microbial substrate availability (Lehmann and Kleber, 2015; Cotrufo et al., 2013). Recent syntheses highlight that soil organic matter dynamics vary across landscapes and cannot be fully understood from bulk measurements alone, underscoring the importance of interpreting chemical indicators—including OM-related metrics and CEC—within their geomorphic and climatic context (Doetterl et al., 2025). Together, organic matter chemistry and CEC shape the “chemical habitat” in which microbes operate and where management practices ultimately determine whether soils retain nutrients and buffer stress or shift toward depletion and instability (Bünemann et al., 2018; Lal, 2016; Sparks, 2003).

Because chemical constraints interact strongly with physical structure and microhabitats, management practices that modify organic inputs, disturbance intensity, and nutrient delivery can guide soil chemistry-microbiome feedbacks toward greater resilience under climate change (Lehmann et al., 2020; Bünemann et al., 2018).

### 4.3 Management determinants: practices shaping habitats and microbiome function

Management acts as a key lever to influence soil physical and chemical constraints, along with the microbiome processes that link them, steering them toward resilience in the face of climate change. By adjusting disturbance intensity (such as tillage and traffic), residue and organic matter inputs, plant cover and rooting patterns, and nutrient delivery, management reshapes microhabitats at the pore scale—such as oxygen and moisture gradients, redox microsites, and substrate accessibility—that govern microbial community assembly and functional activity (Young and Crawford, 2004; Totsche et al., 2018; Bünemann et al., 2018). Since these constraints are interconnected, interventions aimed at improving habitat continuity and resource supply typically enhance functional redundancy and stabilize crucial soil functions like nutrient cycling, aggregation, and water regulation (Delgado-Baquerizo et al., 2016; Banerjee et al., 2018; Lehmann et al., 2020).

Disturbance and organic inputs are key drivers of microbiome-mediated structure and nutrient cycling. Conventional tillage and repeated traffic disrupt aggregates, reduce pore connectivity, and increase compaction risk, which limits oxygen diffusion and can suppress microbial activity and root penetration (Hamza and Anderson, 2005; Beylich et al., 2010). In contrast, reduced disturbance combined with residue retention and organic amendments tends to rebuild aggregation and porosity and increase infiltration and water-holding capacity, conditions that support microbial growth and stabilize functions under heat and drought (Blanco-Canqui and Lal, 2007; Franzluebbers, 2002). Mechanistically, added organic substrates fuel microbial production of extracellular polymeric substances (EPS) and biofilms that promote particle binding, while microbial residues contribute to stable aggregates and longer-term carbon protection (Six et al., 2004; Costa et al., 2018; Lehmann et al., 2020). These changes strengthen habitat buffering and can reduce the sensitivity of microbial processes to drying-rewetting cycles that otherwise destabilize nutrient release and greenhouse gas dynamics (Schimel et al., 2007; Young and Crawford, 2004).

Plant-driven management, including diversity, cover crops, and rotations, influences rhizosphere microbiomes and ecosystem services. Cover crops and diversified rotations increase continuous root inputs and rhizosphere activity, improving soil structure and nutrient retention while supporting microbial functional pathways linked to C-N-P cycling and disease suppression (Schipanski et al., 2014; Bünemann et al., 2018). These practices can enhance microbial functional redundancy and promote beneficial taxa and guilds that contribute to soil aggregation and nutrient-use efficiency, helping stabilize yields under climate variability (Wagg et al., 2014; Delgado-Baquerizo et al., 2016). More targeted “precision microbiome management” approaches are emerging, focusing on context-specific steering of soil, plant, and pest-associated microbiomes using integrated agronomic tools and diagnostics (French et al., 2021). This approach is especially relevant as climate stress increases spatial and temporal heterogeneity in microbial habitats and functions.

Microbiome-informed inputs and integrated fertility strategies can enhance resilience, but their effectiveness depends on context. Biofertilizers and microbial inoculants (e.g., nutrient-mobilizing consortia) can supplement integrated soil fertility management by improving nutrient uptake and fertilizer efficiency, especially where water stress limits diffusion and plant access to sparingly available pools (Vanlauwe et al., 2010; Adesemoye and Kloepper, 2009; García-Berumen et al., 2024). However, successful establishment and persistence depend on the compatibility of the inoculant with local soil structure and chemistry (including pH and salinity) and with plant hosts, as well as reduced disturbance and sufficient organic inputs to maintain functional expression (Busby et al., 2017; Compant et al., 2019). Therefore, management for climate resilience should focus on “habitat-first” strategies (structure, moisture buffering, and organic matter building) and then use microbiome steering (inoculants/consortia, nutrient management timing and forms, and crop-driven rhizosphere selection) to amplify desirable functions rather than expecting universal responses across environments (Lehmann et al., 2020; Bünemann et al., 2018).

Across agroecosystems, soil health results from interconnected feedback loops among physical structure, chemical gradients, and microbiome activity, rather than from any single factor. Pore connectivity and aggregation control oxygen and moisture microhabitats that influence microbial groups, while microbial EPS/biofilms and residues help stabilize aggregates and buffer habitats (Chenu, 1995; Young and Crawford, 2004; Zhang et al., 2024). Similarly, chemically mediated interactions, like rhizosphere acidification through organic acids that mobilize phosphorus, connect plant inputs to microbial function and nutrient absorption (Jones, 1998). These cross-domain processes drive emergent outcomes such as nutrient-use efficiency, water regulation, and resilience under climate extremes (Bünemann et al., 2018; Lehmann et al., 2020; de Vries et al., 2013).

## 5 Management practices for enhancing soil resilience

Management determines whether soils amplify or buffer climate stress because it directly influences soil structure, chemical gradients, and the microhabitats where microbiomes function. Practices that reduce disturbance and increase continuous plant inputs generally improve pore connectivity, moisture buffering, and microbial functional redundancy, thereby stabilizing nutrient cycling and recovery during droughts and heat (Bünemann et al., 2018; Lehmann et al., 2020; Schipanski et al., 2014). In contrast, repeated tillage and traffic increase bulk density, disrupt aggregates, and limit oxygen diffusion, thereby constraining microbial pathways and reducing resilience to extreme conditions (Hamza and Anderson, 2005; Beylich et al., 2010).

Habitat-building practices generate first-order improvements that support beneficial microbial functions. Reducing tillage maintains aggregate structure and pore networks, which support fungal hyphae, EPS/biofilm formation, and microbial residue buildup that stabilize aggregates and enhance water regulation (Six et al., 2004; Costa et al., 2018; Lehmann et al., 2020; Hartmann and Six, 2023). Cover crops and diverse rotations increase rhizodeposition and root exudation, shaping rhizosphere communities toward functions related to nutrient uptake and disease suppression, including competitive exclusion and induced systemic resistance in suppressive soils (Schipanski et al., 2014; Mendes et al., 2011; Busby et al., 2017; Compant et al., 2019). Organic amendments (compost, manure, biochar) alter pH and CEC while providing substrates that increase microbial biomass and enzyme activity; microbial processing of these inputs can be stabilized as mineral-associated organic matter, promoting long-term carbon storage and nutrient buffering (Cotrufo et al., 2013; Lehmann and Kleber, 2015; Lehmann et al., 2020).

Microbiome steering complements habitat building when aligned with local constraints. Integrated Soil Fertility Management (ISFM) combines organic and mineral inputs to synchronize nutrient supply and enhance nutrient-use efficiency (Vanlauwe et al., 2010). Microbial inoculants (e.g., rhizobia, AMF, phosphate-solubilizing bacteria) can boost N and P acquisition through symbioses and via organic-acid and phosphatase-mediated mobilization, effects that are especially important under diffusion limitations during drought (Adesemoye and Kloepper, 2009; Smith and Smith, 2011; García-Berumen et al., 2024). However, inoculant performance is context-dependent and influenced by soil pH, salinity, and microhabitat continuity; therefore, inoculation should be viewed as an amplifier of habitat and resource management rather than a universal substitute (Busby et al., 2017; Compant et al., 2019).

Table 2 summarizes these management levers using a mechanistic logic (constraint → microbiome-mediated mechanism → measurable indicators → functional outcomes) to support interpretation and decision-making under climate stress. This synthesis emphasizes microbiome-relevant mechanisms supported by recent evidence across agroecosystems.

**Table 2:** Management practices and microbiome-relevant mechanisms that enhance soil resilience under climate stress.

Practice (management lever)	Primary constraint targeted (physical/chemical/microhabitat)	Key microbiome-mediated mechanism(s)	Expected functional outcome(s) under climate stress	Measurable indicators	References
Reduced tillage / no-till	Aggregate disruption, pore connectivity loss, compaction risk	Preserves pore network; promotes fungal hyphae and EPS/biofilms; increases aggregate stability via microbial binding and residue-derived substrates	Improved infiltration/retention; greater drought buffering; faster recovery after disturbance	Aggregate stability; bulk density; macroporosity; infiltration; microbial biomass; enzyme activity	Blanco-Canqui and Lal, 2007; Costa et al., 2018; Lehmann et al., 2020; Hartmann and Six, 2023; Six et al., 2004
Traffic control / compaction reduction (incl. grazing management)	High bulk density → O₂ diffusion limits, reduced root penetration	Restores aeration and habitat continuity; avoids shifts toward low-efficiency metabolism under diffusion limitation	Higher microbial activity efficiency; improved root growth; stabilized N cycling	Bulk density; porosity; LLWR; respiration/qCO₂; nitrification/denitrification proxies	Hamza and Anderson, 2005; Beylich et al., 2010; Betz et al., 1998; Totsche et al., 2018
Cover crops / diversified rotations	Low continuous C inputs; weak rhizosphere habitat; erosion risk	Increases rhizodeposition/exudates; recruits beneficial rhizosphere guilds; supports disease suppression and ISR pathways	Higher nutrient retention/use efficiency; improved structure; greater yield stability	SOC/OM fractions; enzyme activities; microbial diversity/network metrics; infiltration; disease suppression indicators	Schipanski et al., 2014; Mendes et al., 2011; Busby et al., 2017; Compant et al., 2019
Organic amendments (compost/manure/biochar)	Low OM, poor buffering (pH/CEC), limited substrate accessibility	Provides C substrates → biomass/enzymes; improves pH buffering/CEC; supports microbial processing → MAOM formation	Greater nutrient buffering; improved water retention via aggregation; long-term C stabilization	SOC fractions (POM/MAOM proxies); CEC; pH; microbial biomass; phosphatase/β-glucosidase	Franzluebbers, 2002; Cotrufo et al., 2013; Lehmann and Kleber, 2015; Lehmann et al., 2020; Doetterl et al., 2025
Integrated Soil Fertility Management (ISFM)	Asynchrony of nutrient supply; low NUE; depleted fertility	Combines organic + mineral inputs to support microbial immobilization–mineralization balance; improves NUE and resilience	Reduced nutrient losses; more stable productivity under variability	Mineral N (NH₄⁺/NO₃⁻); available P; C:N:P; microbial biomass/activity; yield stability	Vanlauwe et al., 2010; Adesemoye and Kloepper, 2009; Cleveland and Liptzin, 2007
Microbial inoculants: rhizobia/PGPR	Nutrient acquisition limitations; stress signaling in rhizosphere	N fixation; phytohormone modulation; siderophores; stress mitigation traits (incl. ISR-associated functions)	Improved root growth and nutrient uptake; resilience under drought/salinity	Nodulation/plant N status; NUE; microbial functional markers; plant performance under stress	Adesemoye and Kloepper, 2009; Lugtenberg and Kamilova, 2009; Compant et al., 2019; French et al., 2021
AMF inoculation / mycorrhizal management	P limitation + water limitation (drylands)	Enhanced P uptake; improved soil exploration; improved plant water relations; contributes to aggregation via hyphal networks	Better drought tolerance; improved P nutrition and yield stability	Plant P status; mycorrhizal colonization; aggregation; plant-available water	Smith and Smith, 2011; Hartmann and Six, 2023
Phosphate-solubilizing microbes / P-mobilization strategies	Low P availability (sparingly soluble pools), diffusion limits under drought	Organic acids + phosphatases mobilize P; improves plant–microbe nutrient acquisition under water stress	Higher P availability; improved productivity in semi-arid contexts	Available P; phosphatase activity; plant P uptake; yield stability	Jones, 1998; García-Berumen et al., 2024; Lei et al., 2025
Disease-suppressive soil management (diversity + rotations + inputs)	Pathogen outbreaks favored by stress; loss of protective networks	Maintains antagonistic guilds; competition/antibiosis; ISR priming in rhizosphere	Reduced disease incidence under heat/moisture extremes; higher stability	Community structure/network metrics; disease incidence; yield stability	Mendes et al., 2011; Busby et al., 2017; Compant et al., 2019
Monitoring & adaptive management (indicator-guided)	Hidden degradation trajectories; delayed detection	Uses biological + physical + chemical indicators to track functional change and adjust practices	Faster corrective action; improved resilience trajectories	Microbial biomass/enzymes; pH/EC; aggregation/infiltration; multi-omics (optional)	Schloter et al., 2018; Nikitin et al., 2022; Bünemann et al., 2018

## 6 Challenges and future directions

Despite significant advances in soil health research, key uncertainties remain about how physical structure, chemical gradients, and microbiome functions interact dynamically under real field conditions and during climate extremes. Closing these gaps is essential to shift from descriptive assessments to predictive, site-specific strategies that reliably improve resilience and maintain productivity under drought, heat, salinity, and flooding (Lehmann et al., 2020; Bünemann et al., 2018).

A key limitation is that many studies still treat soil physical, chemical, and biological domains as separate topics rather than as a connected system influenced by pore-scale constraints, resource diffusion, and microbial functional responses. However, climate impacts are often mediated through microhabitats (oxygen and moisture heterogeneity, redox microsites, and substrate accessibility) that link structure and chemistry to shifts in microbial guilds and biogeochemical rates (Young and Crawford, 2004; Totsche et al., 2018). Future research should therefore focus on integrative frameworks that explicitly connect: (i) stressors, (ii) microhabitat constraints, (iii) microbiome functional pathways, and (iv) emergent functions and outcomes, using measurable indicators across domains (Lehmann et al., 2020).

Traditional indicators (e.g., bulk density, pH, total organic matter) remain valuable, but they can overlook the spatial-temporal dynamics that control function during extremes. Progress will require functional and process-relevant measurements, such as enzyme systems, functional genes/transcripts, and redox-sensitive pathway indicators, interpreted alongside pore network properties and moisture/oxygen regimes (Baveye et al., 2016; Schloter et al., 2018; Nikitin et al., 2022).

Emerging methods can overcome these limitations when integrated into coherent workflows. For example, X-ray microtomography (structure), combined with in situ moisture/redox sensing (microhabitat constraints) and metagenomics/metatranscriptomics (functional potential and expression), can test mechanistic hypotheses about why specific guilds (e.g., nitrifiers versus denitrifiers) dominate during drought-rewetting or flooding cycles and how that impacts nitrogen losses and recovery trajectories. Isotopic labeling and spectroscopy further enable tracking of carbon stabilization and nutrient fluxes that are strongly mediated by microbial processing and mineral association (Cotrufo et al., 2013; Lehmann and Kleber, 2015). However, these tools are still underused due to high costs, limited standardization, and barriers to adoption in low-resource settings, highlighting the need for harmonized protocols and tiered monitoring approaches (Baveye et al., 2016; Totsche et al., 2018). Table 3 summarizes emerging tools that enable cross-domain integration by linking pore-scale structure and microhabitat dynamics with microbiome functional potential and expression under climate stress.

**Table 3:** Emerging tools to monitor soil health dynamics and microbiome-mediated processes across scales.

Tool / approach	Cross-domain variable captured	What mechanism it tests (example)	Typical decision enabled	Main limitation(s)	References
Metagenomics / amplicon profiling	Functional potential + community structure	Functional capacity for N/P cycling, stress traits	Baseline diagnosis; monitoring shifts after management	Cost; bioinformatics; inference not expression	Fierer, 2017; Schloter et al., 2018; Nikitin et al., 2022
Metatranscriptomics	Real-time functional expression	Drought/heat induction of stress-response pathways; shifts in enzyme systems	Identify active pathways under extremes	High cost; sampling sensitivity; standardization	Bei et al., 2023; Ramond et al., 2022
X-ray microtomography / 3D imaging	Pore architecture + connectivity	Microhabitat constraints (O₂ diffusion, microsites) shaping guilds	Identify compaction/structure limits; guide tillage/traffic decisions	Lab-based; expensive; scaling	Totsche et al., 2018; Golparvar et al., 2021; Young and Crawford, 2004
Spectroscopy (FTIR/NIRS) + SOM fractionation	SOM quality + stabilization pathways	Microbial processing → MAOM formation vs. labile turnover	Track carbon stabilization; amendment selection	Calibration needed; interpretation	Lehmann and Kleber, 2015; Cotrufo et al., 2013; Lehmann et al., 2020
Stable isotope probing / isotopic labeling	Process rates + substrate flow	Link active taxa/pathways to C/N transformations	Validate mechanisms; refine models	Resource intensive; limited throughput	Liu et al., 2024; Ramond et al., 2022
Viral metagenomics / soil viromics	Viral diversity, virus-host interactions, auxiliary metabolic genes	Viral shunt, host mortality, and viral modulation of C—N-P cycling	Identify overlooked viral controls on microbial turnover and nutrient cycling	High methodological sensitivity; incomplete viral databases; difficult host assignment; limited field standardization	Emerson, 2019; Sun et al., 2023; Graham et al., 2024; Liu et al., 2024
In situ sensors (moisture, temperature, redox/Eh)	Microhabitat dynamics over time	Drought–rewetting pulses; flooding redox transitions	Early warning; irrigation/drainage timing	Spatial coverage; maintenance	Baveye et al., 2016; Schimel et al., 2007; Evans and Wallenstein, 2014

Although meta-omics approaches provide unprecedented resolution for linking microbial communities with soil functions, their routine field-scale use remains limited (Fierer, 2017; Schloter et al., 2018; Nikitin et al., 2022). Metagenomics can identify functional potential, but it does not necessarily indicate which pathways are active under a given stress condition (Fierer, 2017; Schloter et al., 2018). Metatranscriptomics and metaproteomics provide closer approximations of functional expression, but they are highly sensitive to sampling time, RNA/protein preservation, extraction protocols, bioinformatic pipelines, and short-term fluctuations in soil moisture, temperature, and redox conditions (Schloter et al., 2018; Bei et al., 2023). In addition, high costs, incomplete reference databases, limited cross-laboratory standardization, and difficulties in scaling from microsites to field plots restrict their operational use in soil health monitoring (Baveye et al., 2016; Schloter et al., 2018; Nikitin et al., 2022). Therefore, meta-omics should be integrated into tiered frameworks: low-cost physical, chemical, and biological indicators can support routine monitoring, while targeted meta-omics, isotopic tracing, and pore-scale imaging can be used to validate mechanisms, identify active pathways, and refine predictive models under climate stress (Baveye et al., 2016; Schloter et al., 2018; Nikitin et al., 2022).

Soil viruses also represent an important frontier for cross-domain soil health research. Although bacteria and fungi have received most attention in microbiome-based assessments, viral communities may regulate microbial turnover, nutrient cycling, and functional resilience through host lysis, virus–host coevolution, and auxiliary metabolic genes. Integrating soil viromics with metagenomics, stable-isotope approaches, redox/moisture monitoring, and pore-scale characterization could help determine when viral pathways become functionally important under drought–rewetting, flooding, salinity, or nutrient stress. However, routine incorporation of viral indicators into soil health monitoring remains limited by incomplete viral reference databases, methodological sensitivity, uncertain host assignment, and limited standardization across soils and climates (Emerson, 2019; Sun et al., 2023; Graham et al., 2024; Liu et al., 2024).

Soil animals should also be integrated more explicitly into future microbiome-based soil health frameworks. Although this review focuses primarily on microbial mechanisms, organisms such as nematodes and earthworms can strongly influence microbial habitats, nutrient cycling, aggregation, and plant performance (Brussaard et al., 2007; Bardgett and van der Putten, 2014). Earthworms modify soil structure through burrowing, casting, organic matter fragmentation, and the creation of biopores that alter aeration, water infiltration, and microbial microsites (Lavelle et al., 2006; Brussaard et al., 2007). Nematodes regulate microbial communities through grazing and trophic interactions, influencing mineralization, disease suppression, and nutrient availability (Ferris et al., 2001; de Vries et al., 2013). Therefore, soil animal–microbe interactions represent an important frontier for understanding how biological networks regulate soil resilience under climate stress (de Vries et al., 2013; Bardgett and van der Putten, 2014).

Another major gap is the limited number of long-term field experiments that measure how management changes reshape the coupled soil system over time, including delayed responses in soil structure, organic matter pools, and microbiome composition and function. Long-term studies demonstrate that management can alter soil trajectories, but outcomes depend heavily on soil type, climate, and baseline degradation levels (Parras-Alcántara et al., 2016). Translating microbiome-based interventions into practice also requires accounting for socioecological constraints: locally feasible monitoring, economic viability, and culturally appropriate management options for smallholders and traditional systems.

Finally, microbiome interventions (inoculants, consortia, microbiome steering) will be most effective when designed around habitat compatibility (pH, salinity, pore continuity, and moisture buffering) and functional targets (e.g., P mobilization, N retention, aggregation/EPS, stress tolerance), rather than taxonomy alone. Developing predictive, cross-domain decision tools and composite indicators that connect management levers to functions and outcomes remains a priority (García-Berumen et al., 2024; Wen et al., 2025).

## 7 Conclusions

Soil health under climate change is governed by tightly coupled feedbacks among soil structure, chemical gradients, and microbiome functions. A central insight of this review is that climate impacts are often mediated by pore-scale microhabitats (oxygen and moisture heterogeneity, redox microsites, and substrate accessibility) that reorganize microbial functional guilds and regulate aggregation, organic matter turnover, and C–N–P cycling. These mechanisms jointly determine emergent soil functions relevant to resilience and yield stability.

From a practical perspective, the evidence indicates that microbiome-based interventions are most robust when “habitat-first” strategies (improving structure, moisture buffering, and chemical balance) are paired with context-dependent microbiome steering (diverse rotations/cover crops, organic inputs, and targeted inoculants), guided by cross-domain indicators rather than single metrics.

Key knowledge gaps and priorities include: (i) long-term, field-scale experiments quantifying time-lagged responses in structure, chemistry, and microbiome function during extreme events; (ii) standardized, tiered monitoring frameworks that pair low-cost indicators with targeted, high-resolution measurements to validate mechanisms; (iii) predictive models that integrate microhabitat dynamics with microbiome function to support site-specific decision-making; and (iv) the integration of soil viruses into microbiome-based soil health frameworks, because virus–host interactions and viral auxiliary metabolic genes may represent overlooked controls on microbial turnover and nutrient cycling under climate stress. Addressing these priorities will strengthen climate-smart soil management and accelerate the development of resilient and sustainable food systems.

## Funding

This research did not receive any specific grants from funding agencies in the public, commercial, or not-for-profit sectors.

## Declaration of competing interest

Given his role as Associate Editor, Sergio de los Santos-Villalobos had no involvement in the peer-review of this article and had no access to information regarding its peer-review. Full responsibility for the editorial process for this article was delegated to David Ojcius.

The authors declare that the work was conducted in the absence of any commercial or financial relationships that could be construed as a potential conflict of interest.

## Data Availability

The datasets used and/or analyzed during the current study are available from the corresponding author upon reasonable request.
